# Revealing the impact of social circumstances on the selection of cancer therapy through natural language processing of social work notes

**DOI:** 10.1093/jamiaopen/ooae073

**Published:** 2024-10-11

**Authors:** Shenghuan Sun, Travis Zack, Christopher Y K Williams, Atul J Butte, Madhumita Sushil

**Affiliations:** Bakar Computational Health Sciences Institute, University of California, San Francisco, San Francisco, CA 94143, United States; Bakar Computational Health Sciences Institute, University of California, San Francisco, San Francisco, CA 94143, United States; Division of Hematology/Oncology, Department of Medicine, University of California, San Francisco, San Francisco, CA 94143, United States; Bakar Computational Health Sciences Institute, University of California, San Francisco, San Francisco, CA 94143, United States; Bakar Computational Health Sciences Institute, University of California, San Francisco, San Francisco, CA 94143, United States; Center for Data-driven Insights and Innovation, University of California, Office of the President, Oakland, CA 94607, United States; Department of Pediatrics, University of California, San Francisco, San Francisco, CA 94158, United States; Bakar Computational Health Sciences Institute, University of California, San Francisco, San Francisco, CA 94143, United States

**Keywords:** natural language processing, social work notes, social determinants of health, cancer therapy, selection of cancer therapy

## Abstract

**Objective:**

We aimed to investigate the impact of social circumstances on cancer therapy selection using natural language processing to derive insights from social worker documentation.

**Materials and Methods:**

We developed and employed a Bidirectional Encoder Representations from Transformers (BERT) based approach, using a hierarchical multi-step BERT model (BERT-MS), to predict the prescription of targeted cancer therapy to patients based solely on documentation by clinical social workers. Our corpus included free-text clinical social work notes, combined with medication prescription information, for all patients treated for breast cancer at UCSF between 2012 and 2021. We conducted a feature importance analysis to identify the specific social circumstances that impact cancer therapy regimen.

**Results:**

Using only social work notes, we consistently predicted the administration of targeted therapies, suggesting systematic differences in treatment selection exist due to non-clinical factors. The findings were confirmed by several language models, with GatorTron achieving the best performance with an area under the receiver operating characteristic curve (AUROC) of 0.721 and a Macro F1 score of 0.616. The UCSF BERT-MS model, capable of leveraging multiple pieces of notes, surpassed the UCSF-BERT model in both AUROC and Macro-F1. Our feature importance analysis identified several clinically intuitive social determinants of health that potentially contribute to disparities in treatment.

**Discussion:**

Leveraging social work notes can be instrumental in identifying disparities in clinical decision-making. Hypotheses generated in an automated way could be used to guide patient-specific quality improvement interventions. Further validation with diverse clinical outcomes and prospective studies is essential.

**Conclusions:**

Our findings indicate that significant disparities exist among breast cancer patients receiving different types of therapies based on social determinants of health. Social work reports play a crucial role in understanding these disparities in clinical decision-making.

## Objective

Clinical decisions biased by social disparities lead to significant discrepancies in outcome and pose significant public health concerns.^1–3^ Clinical decisions are influenced not only by clinical criteria but also by non-clinical factors such as race, gender, perceived financial stability, and more, which are collectively referred to as social determinants of health (SDOH).^4–6^ There is growing evidence that many minority groups are less likely to receive standard of care.^6–10^ One pressing example is the decision to initiate anti-neoplastic treatments, which are becoming increasingly expensive and associated with financial toxicities.^9^ While new, targeted agents often are better tolerated and more effective than previous treatments, they can come with a high price tag not always fully covered by insurance, leaving clinicians with a moral decision when balancing efficacy and cost. Financial constraints are but one example of factors that can potentially influence the treatment decision.^10^

Our results indicate a potential association between specific features within social work (SW) clinical documentation and the choice of expensive, targeted therapy prescription for patients with breast cancer. Using a pre-trained Bidirectional Encoder Representations from Transformers (BERT) model, we showed that the unstructured SW notes, without detailed diagnostic or therapeutic information, can predict whether targeted therapy was prescribed for a given patient. Moreover, we developed a hierarchical language model for prediction over long sequences of clinical notes and successfully increased the predictability of the outcome. To understand which SDOH factors are used by the model for prediction, we measured the importance of SDOH factors by deleting words belonging to specific SDOH topics. Several critical contributors emerged, including socio-economic factors, abuse history, and risk of death. Our findings demonstrate that SW notes can reveal the impact of a patient's social environment on medical treatment prescription without requiring expensive and time-consuming manual annotation. Our hierarchical modeling approach will inform the development of models capable of leveraging multiple clinical notes for prediction.

## Background and significance

A growing body of evidence indicates that SDOH factors significantly impact patient health and behaviors.^5^^,^^6^^,^^11^^,^^12^ However, SDOH factors not only affect patients but also influence the clinical decision-making process recommended by physicians.^4^ Ideally, clinical decision-making should be rooted in evidence-based practices, cognizant of the complex interaction between a patient’s background and SDOH that could affect both their trust in the medical system and their overall disease trajectory. In reality, though, physicians are inevitably influenced by a wide range of non-clinical factors, with many of these non-clinical factors rooted in unconscious bias.^13^^,^^14^ Previous research showed that clinical management decisions can be influenced by socioeconomic status,^8^ race,^15^ gender,^16^ adherence to treatment,^17^ patient behavior,^18^ attitude,^19^ and even physician personal characteristics.^20^

Although it is well-known that SDOH-related, non-medical factors are crucial contributors to health and clinical outcomes, extracting non-medical and social factors from electronic medical records remains challenging. While information such as smoking, alcohol, and primary insurance status is increasingly accessible in structured fields, many social factors that are increasingly recognized as being important to successful treatment are either not captured or are not a focus of structured physician documentation. Various aspects that physicians consider, including patient personalities, preferences, faith, concerns, professional interactions, family support, and living situations, can often be missing or improperly addressed within physician notes.^4^ Given the lack of structured data, traditional association analysis is not feasible, emphasizing the need for innovative approaches.

Compared to general clinical documentation, notes written by social workers (SW notes) contain comprehensive social information.^21^^,^^22^ Social workers are professionals who specialize in navigating a patient through the barriers that may interfere with receiving adequate medical care.^23^^,^^24^ They can evaluate the many aspects of patients’ life outside of medicine that can impact their ability to receive treatment. The unique and rich content of SW notes about SDOH makes them invaluable; however, transforming this information into structured, categorical data for traditional analysis would be prohibitively time-consuming and costly, potentially wasting resources without a sophisticated design for annotation.

Instead, using transformer-based predictive models allows us to bypass the extensive need for manual annotation by employing an end-to-end framework that learns patterns directly from the data. Although these models do not establish causality—as traditional statistical methods might—strong correlations identified by the predictive models still underscore the significant insights that can be derived from understanding how SDOH influences clinical decision-making.

## Materials and methods

### Study design and cohort selection

**Figure 1.**
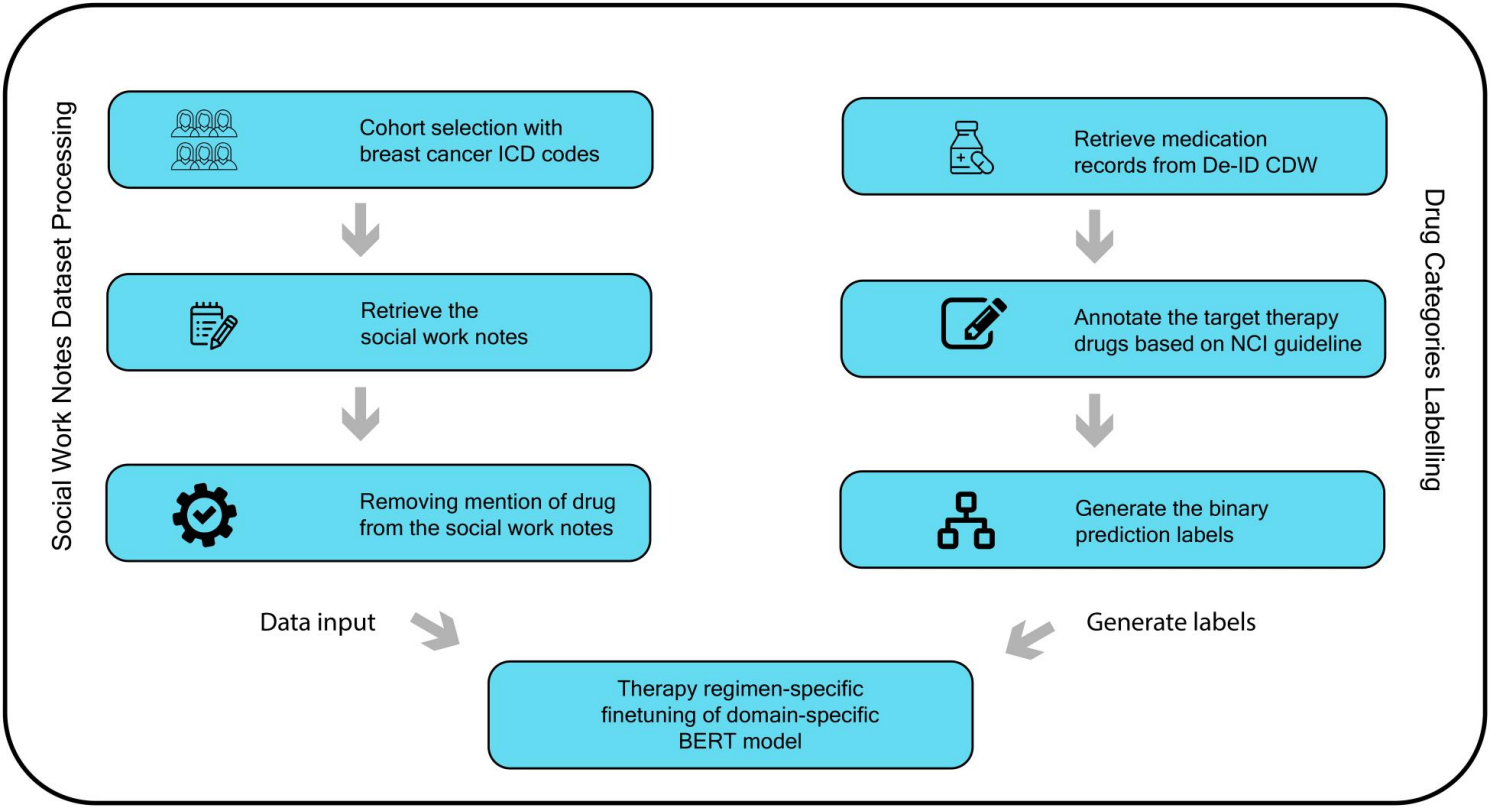
The overall workflow. We implement an end-to-end BERT-base classification model to predict the category of treatment administration for breast cancer patients at UCSF. We first retrieved the patients’ social work notes from UCSF de-identified Caboodle Data Warehouse (DeID-CDW) between 2012 and 2021. We then annotated whether an individual patient has ever received targeted therapy based on the Targeted Cancer Therapies Fact Sheet from National Cancer Institute. In this manner, we obtained 2496 patients, of which 70% received targeted therapy. The dataset was further split into 8:1:1 ratio, corresponding to training, validation, and test sets.

This study used a deidentified clinical note corpus at UCSF available within the UCSF Information Commons. The research was conducted under the IRB #18-25163. Our corpus included the deidentified social work notes of all patients treated at UCSF for breast cancer between 2012 and 2021 (Figure 1)^25–27^. Breast cancer diagnosis was identified using the ICD9 code 174 and the ICD10 code C50 through the UCSF Clinical Data Warehouse. We obtained 2496 patients matching these codes, with available social work reports. We then retrieved the medications ordered or prescribed for these patients, then categorized these as “targeted therapy” medications or not based on the definitions in the *Targeted Cancer Therapies Fact Sheet from National Cancer Institute.*^28^ Patients in the cohort who received targeted therapy at least once were categorized into the “*Targeted therapy administered*” group (TT-Yes); patients who did not receive any targeted therapy were categorized into the “*Targeted therapy not administered*” group (TT-No). Though we are working with social work notes, we still found that drug information was mentioned in less than 10% of the overall social work notes. To prevent information leakage, we masked the drug information prior to any further processing. Specifically, in expressions like “*Tamoxifen was administered to the patient*,” we replaced the drug name, here *Tamoxifen*, with the word “*drug.*” The complete list of drug names that were masked are included as Table S11.

### Deep learning models for sentence classification

We used the latest and the longest social work note per patient to predict cancer therapy selection. Patient notes were randomly split in an 8:1:1 ratio into training, validation and test sets. We trained our algorithm on the training set, using the early stopping approach to help with parameter tuning on the validation set. We ran our algorithm 5 times for each model and evaluated the model performance using the validation set. The cross-entropy loss function was used for optimization. After training and hyperparameter tuning, the model was tested on the held-out test set to compute model performance. Median scores over 5 runs are reported here.

We compared several biomedical BERT models in this research, including: GatorTron-OG,^29^ a Megatron BERT model pre-trained on de-identified clinical notes from the University of Florida, the UCSF-BERT model,^30^ which is a cased BERT model pre-trained on the UCSF clinical notes publicly, SciBERT,^31^ ClinicalBERT,^32^ BioLM,^33^ and Biomed-Roberta.^34^ All of these models have been pre-trained on a large corpus of scientific texts, PubMed, PMC, and/or clinical notes from the MIMIC-III corpus.^35^ We fine-tuned each of these models for the classification task.

To rule out the possibility of finding results at random, we implemented 3 distinct dummy classifiers as a control. Dummy (Prior): This strategy always predicts the most frequent class in the training set. Dummy (Stratified): This strategy generates predictions by respecting the class distribution of the training set. It randomly predicts class labels based on the distribution of the training set. Dummy (Uniform): This strategy generates predictions uniformly at random.

### Evaluation metrics

Model evaluation results were reported for the testing dataset only. For the classification task, area under the receiver operating characteristic curve (AUROC), F1 score, precision, and recall metrics are reported. In order to address the issue of data imbalance, which can impede the interpretation of model performance, we used macro-averaged format for F1, precision, and recall score. F1 score is the harmonic mean of precision and recall.

Notably, macro-averaged computation uses the arithmetic mean of all the per-class scores, which provides equal weight to all the classes. We used *sklearn.metrics* from the scikit-learn python package for programming.^36^

### Constructing the BERT-MS model

**Figure 2.**
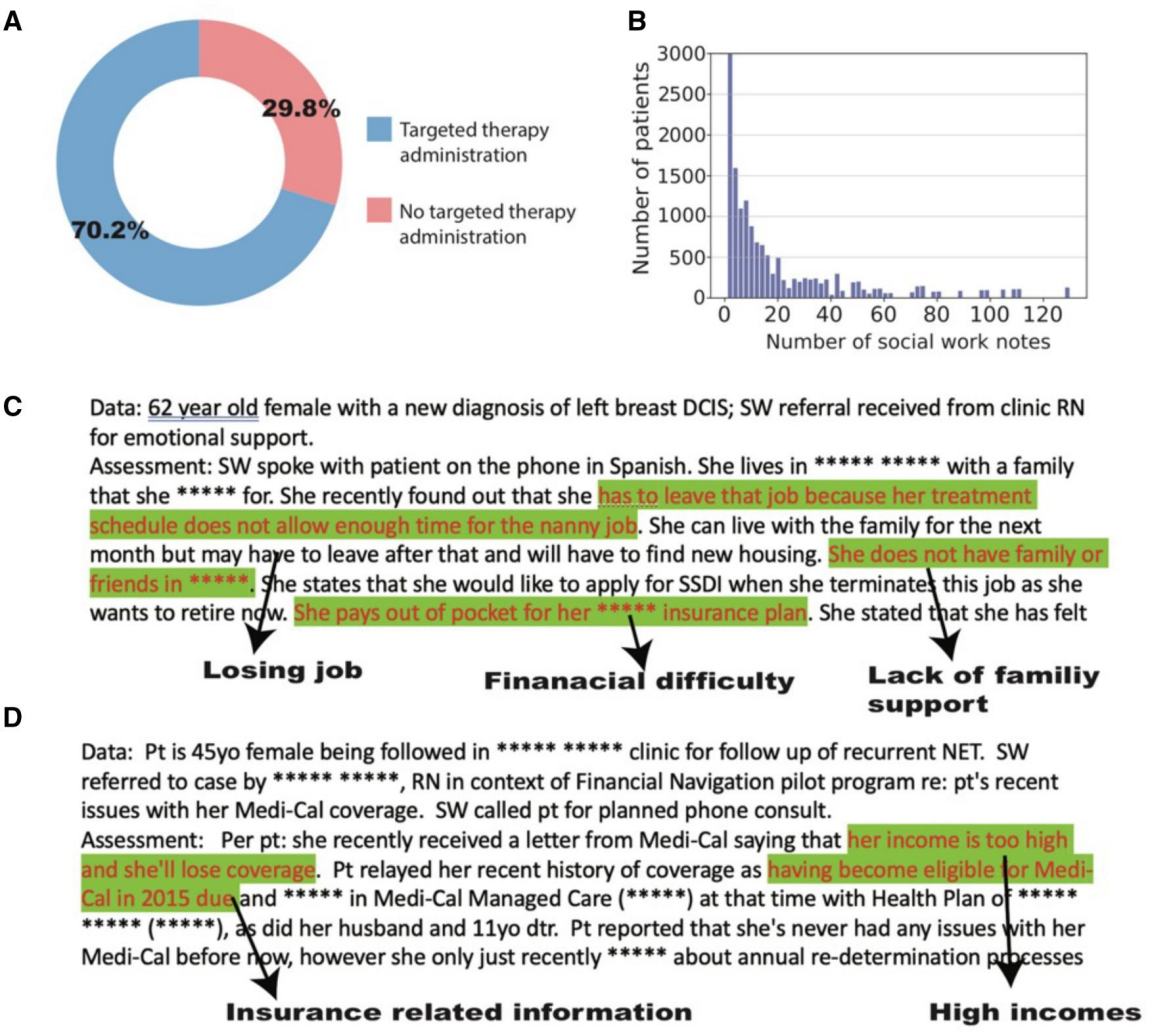
Data exploration on social work notes. (A) Pie chart showing the different proportions of patients in the two categories. (B) Histogram showing the number of notes for the individual patients (mode = 2, mean = 22, median = 11). (C) Example deidentified social work notes. Top: Example patient who did not have any targeted therapy administration. Bottom: Example patient who received at least one dose of targeted therapy.

**Figure 3.**
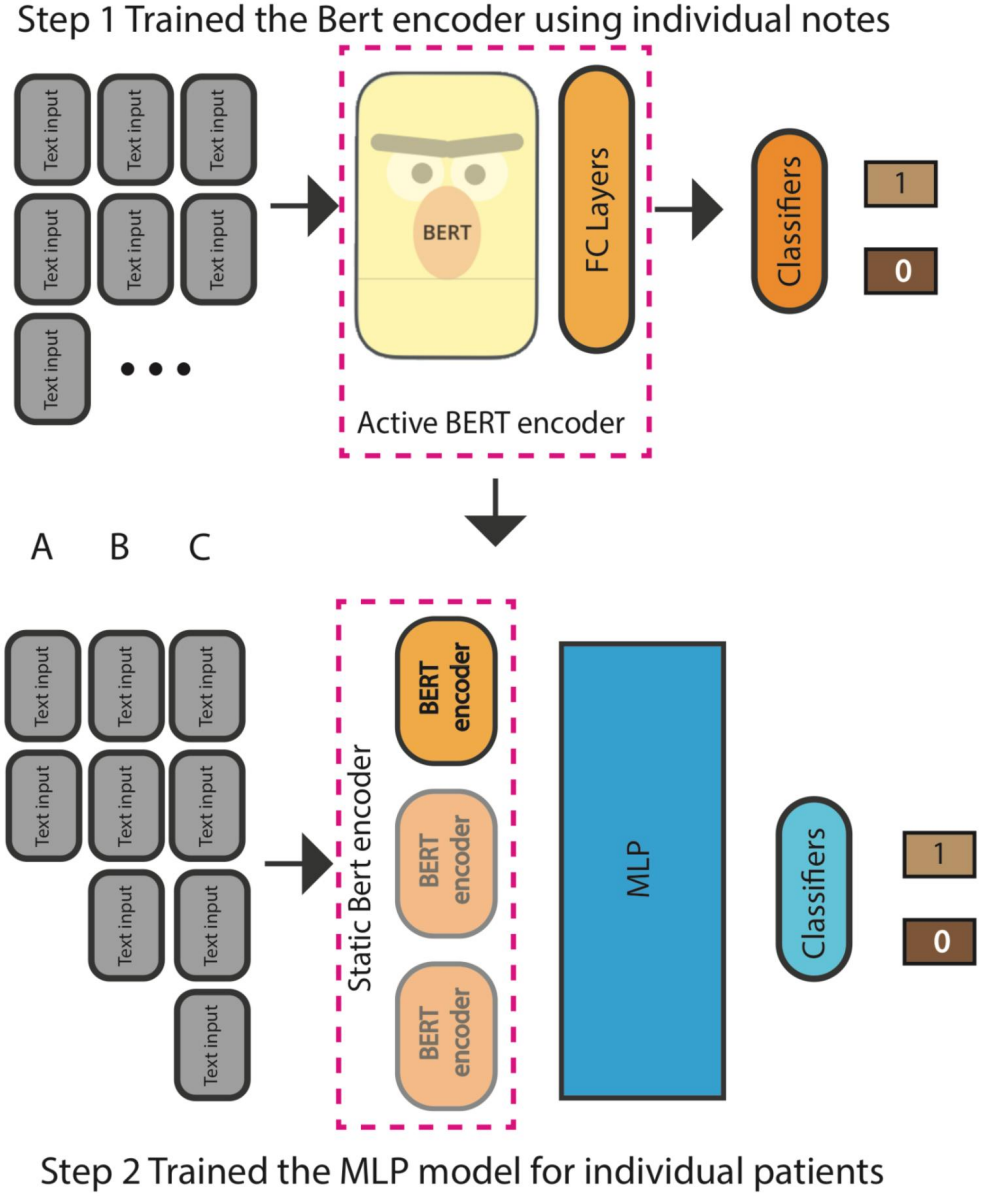
Illustration of BERT-MS-n model. To use long sequences of clinical notes for prediction, we built a hierarchical BERT model (BERT-MS), where the first step divides a long sequence of notes into multiple independent instances and then trains the single BERT classifier on the individual chunks in the training set. In the second step, we concatenate the BERT representations of all notes of the same patient and further fit them into a multilayer perceptron for the training. FC = fully connected layer; MLP = multi-layer perceptron.

Although most patients in our dataset have several relevant SW notes (median = 11, Figure 2B), the BERT models used for classification are unable to accept more than a maximum of 512 tokens, which cannot handle more than one social work note piece. We were interested in knowing whether integrating more notes and thus more information about a patient’s social history would improve the prediction. However, retraining a language model with an input length several times longer would take considerable time and computation resources and is impractical in an academic environment.^37^ Consequently, we developed a multistep, hierarchical BERT model that can integrate several notes named MS-n, where *n* refers to the maximum number of notes allowed by the model (Figure 3).

The MS-n model was trained in 2 steps (Figure 3 and Algorithm S1). First, all clinical notes for a single patient were treated as independent instances for phase 1 fine-tuning. Each note for a single patient was assigned the same binary patient-level label indicating whether targeted therapy was administered to the patient. The BERT model was fine-tuned in this setup and the validation loss was computed for backpropagation. Consequently, in the second phase, intermediate note-level representations were extracted from the resulting model of phase 1 finetuning and concatenated for phase 2 finetuning. The phase 2 BERT model was initialized with these concatenated note-level representations, the intermediate layer weights were frozen, and the classification layer of the model was fine-tuned further. Phase 1 fine-tuning was critical because it could extract the lower-dimensional hidden representations of each note. In this manner, we were able to train a hierarchical language model that can integrate n-fold information without expending the model parameter n-folds. We built several UCSF BERT-MS-n models including MS-3, MS-5, MS-8, MS-10, that correspond to the use of at most 3, 5, 8, and 10 notes.

### Feature importance analysis

To understand which SDOH factors are used by the model for prediction, we used feature ablation methods to measure the importance of different SDOH factors.^38^ We examined the effect on model performance of removing keywords associated with the following topics: Mental health, Family, Consultation/Appointment, Group session, Risk of death, Clinician/Hospital/Medication, Living condition/Lifestyle/Social support, Telephone encounter/Online communication, Abuse history (all forms), and Insurance/Income. These categories, and keywords associated with each category, were selected following the LDA topic modeling analysis as described by Sun et al^39^ (Table S5). Specifically, we removed a set of words belonging to each SDOH topic iteratively from the test set only and compared the decrease in model performance represented by the decrease in F1 score. We conducted these experiments on MS-5 model which has the best predictive performance.

To account for differences in the prevalence of various topics mentioned across patients (eg, 96% of notes contained keywords in the “Social support” topic whereas only 10% of notes relate to the “Risk of death” category), we normalize the importance of each topic by their frequency. We present both the raw feature important score and the important score normalized by topic frequency in Figure S2.

## Results

### Patients structured characteristics and their social work notes

We identified 2496 patients with breast cancer with available deidentified social work notes (Figure 1); 97.9% of patients were female and 2.1% were male. There were 59.7% White/Caucasian patients, 18.1% Asian, 10.1% Hispanic/Latino, 6.5% Black/African, and 15.7% Other (Table S1). No obvious difference was observed when comparing the demographic information between patient with and without social work notes except an increase proportion of Asian population (Table S9). Overall, 70% of patients in the cohort received targeted therapy at least once [“*Targeted therapy administered”* group] (TT-Yes), compared to 30% of patients who did not receive any targeted therapy [“*Targeted therapy not administered”* group] (TT-No).

First, we explored whether SDOH information within structured data alone could stratify these patients. For the 2496 patients identified, we found information regarding demographics, marriage status, and smoking history was present, but data on patient financial status, education level, and other important SDOH were absent from the structured data. Machine learning-based approaches leveraging all available demographic information, marriage status and smoking history failed to predict the administration of targeted therapy in patients (Table S3), which is not surprising given the sparsity of the available data as well as the complexity of the task.

In contrast, our prior research has demonstrated that social work notes possess a wealth of information relating to SDOH, including details on frequently discussed topics such as mental health, insurance status, and family support (Figure 2C and D).^39^ This qualitative observation suggested that social work notes encompass a wealth of SDOH factors, highlighting the challenges associated with extracting these multifaceted and often subtle social determinants from unstructured text. The complexity of these notes, with no standardized reporting or consistent terminology across different documents, makes traditional data extraction methods inadequate. Consequently, we employed advanced pre-trained language models, which are adept at identifying and interpreting the nuanced expressions of SDOH embedded within the free-text format of the notes.

### GatorTron-OG outperforms other language models in predicting therapy

We fine-tuned several pre-trained biomedical BERT models to predict the targeted therapy administration directly from the social work notes of breast cancer patients.^29–34^^,^^40^ Given that the maximum sequence length supported by a regular BERT model is 512 tokens, we used the longest note for each patient to maximize the amount of information available for classification. Table 1 shows the prediction performance of different deep-learning classification models. To ensure that related clues or other explicit medical information were not utilized in the prediction, we additionally quantified model performance on a subset of notes that do not mention any drugs. This approach achieved similar performance, demonstrating the reliability of masking the drug names (Table S7). GatorTron-OG achieved the best result with a Macro F1 of 0.616 and AUROC score of 0.721. UCSF-BERT also held good classification performance with a Macro F1 of 0.599 and AUROC score of 0.675, although it did not outperform the GatorTron-OG model. This can be attributed to the fact that GatorTron model is larger in size and is trained on a larger cohort of clinical data. RoBERTa models (BioLM and Biomed-Roberta) performed generally better than BERT-base models (SciBERT, ClinicalBERT) potentially because of their dynamic masking strategy during pre-training such that the masked token changes during each training epoch.^31^ This suggests that pretraining BERT-based models with clinical data can be helpful for achieving superior performance on domain-specific tasks. We also ran our tasks on 3 random baseline models, each of which ruled out the random performance from different perspectives (see “Materials and methods”**)**. Our model significantly outperformed the random baselines (Table 1).

**Table 1.** Model performance of different classifiers. GatorTron-OG achieved superior performance in AUC, MACRO F1, and MACRO RECALL. Results from basic machine learning models are based on SDOH-related structured tabular data for prediction.

Model	AUC	MACRO F1	MACRO PRECISION	MACRO RECALL
Gatortron-OG	0.721	0.616	0.624	0.611
UCSF BERT	0.675	0.599	0.604	0.596
ClinicalBERT	0.627	0.578	0.584	0.576
SciBERT	0.616	0.532	0.606	0.533
BioLM	0.671	0.583	0.615	0.580
Biomed-RoBERTa	0.667	0.584	0.592	0.581
Dummy (prior)	0.500	0.412	0.350	0.491
Dummy (stratified)	0.504	0.525	0.529	0.603
Dummy (uniform)	0.500	0.509	0.522	0.602
KneighborsClassifier	0.497	0.491	0.496	0.497
SVM Classifier	0.500	0.434	0.383	0.500
RandomForest	0.519	0.483	0.592	0.517
GradientBoosting	0.509	0.458	0.635	0.509

### Integrating multiple clinical notes for prediction

Given that the median number of clinical social notes per patient in our cohort is 11, we built several multi-step (MS-n) models including MS-3, MS-5, MS-8, MS-10, allowing the analysis of up to 3, 5, 8, and 10 notes respectively. We used UCSF-BERT for this because it is smaller in size, and hence has lower training complexity than the GatorTron-OG model, while having comparable performance. Table 2 compares the prediction performance of UCSF BERT_MS-n models with the UCSF BERT model using a single social work note. Generally, the UCSF BERT_MS-n models achieved better results, demonstrating the advantage of incorporating more clinical notes.

**Table 2.** BERT MS model achieved superior performance in AUC, MACRO F1, as well as MACRO RECALL.

	AUC	MACRO F1	MACRO PRECISION	MACRO RECALL
UCSF BERT	0.675	0.599	0.604	0.596
UCSF BERT MS-3	0.707	0.620	0.660	0.612
UCSF BERT MS-5	0.702	**0.624**	0.637	0.615
UCSF BERT MS-8	**0.718**	0.623	0.645	**0.616**
UCSF BERT MS-10	0.706	0.596	**0.665**	0.594

Bold indicates highest performance.

### Identifying the SDOH factors that influence model decisions

To explore the role different SDOH factors may have in predicting utilization of targeted therapy, we assessed the importance of SDOH factors by feature ablation methods (see “Materials and methods”). The 11 topics that we tested were mentioned with varying frequency in the social work notes (Figure S1). The notes belonging in each topic have similar class proportions: 70% “TT-Yes” group and 30% “TT-No” group. Of note, simple machine learning frameworks leveraging the presence of SDOH topics as binary features were not sufficient to predict the administration of targeted therapy (Table S8).

**Figure 4.**
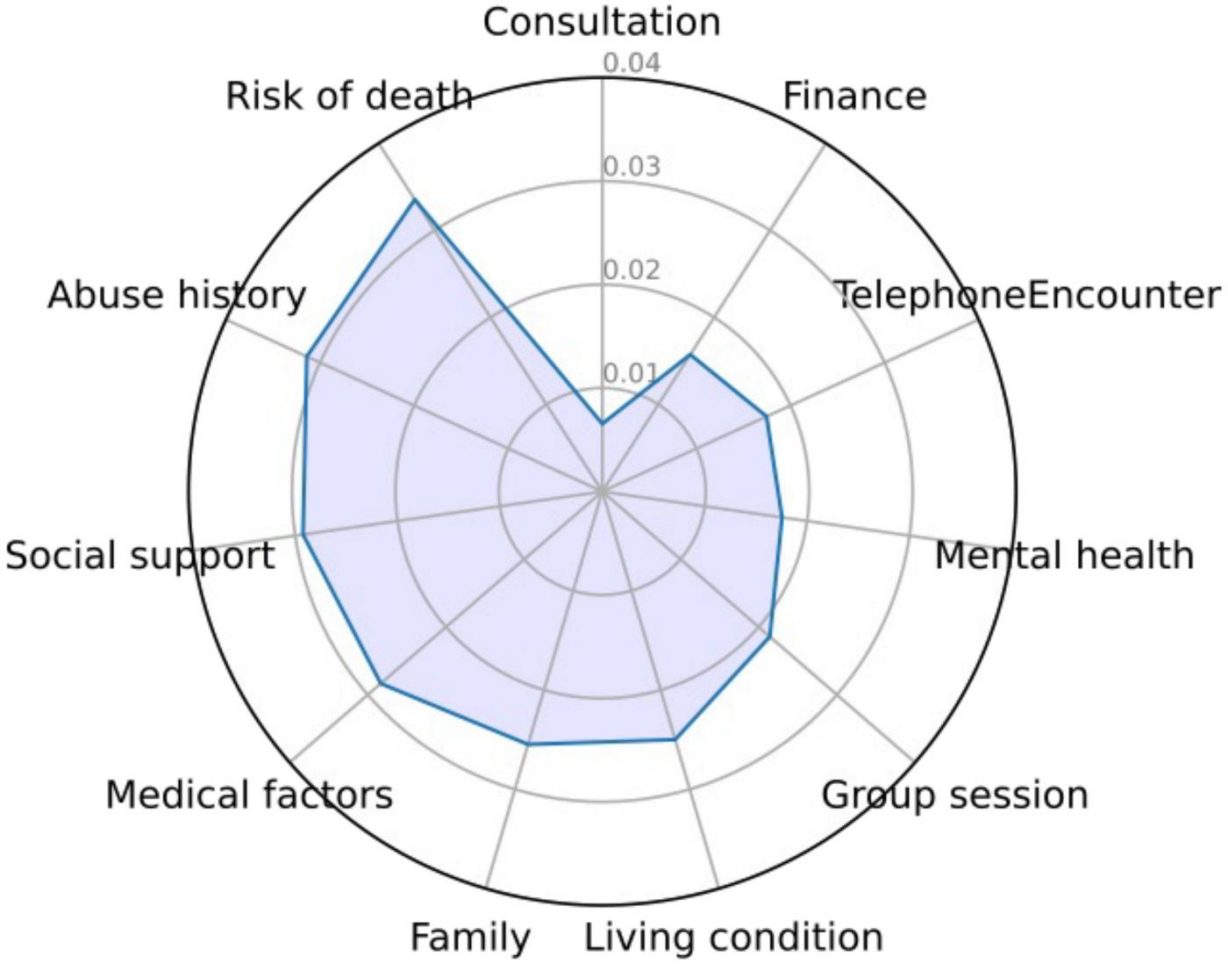
Feature importance analysis for SDOH factors in ablation study. The radar chart shows the feature importance of SDOH topics, represented by the decrease of F1 score.

We identified several SDOH topics, including *Abuse History*, *Risk of Death*, and *Social Support,* as the most significant influencers that the model leveraged in the prediction task (Figure 4**)**. For example, looking at the notes that mentioning about *Abuse History* (Figure S3) we found detailed descriptions of patients' past experiences with domestic violence and verbal abuse, which provided critical context influencing the model's predictions. In one case, a patient described ongoing verbal abuse from an in-law, impacting her mental health and possibly her access to consistent care. Another note highlighted a patient's history of physical abuse by her husband, including recent incidents, which may affect her emotional stability and treatment adherence. These nuanced details about abuse history underscore how deeply personal and sensitive information can inform the model's understanding of a patient's social context and its impact on their treatment choices. None of this information, however, is reflected in the structured data.

Other SDOH topics such as *Family*, *Living Condition* also had obvious impact in model decision making. However, besides the broad topic area “medical factors,” the common topics relating to medical aspects, *Mental Health* and *Group Session*, had a lower influence on the model prediction. As the neutral control, topic *Consultation* and *TelephoneEncounter* played a less important role in the prediction task. Consultation notes typically covered routine medical consultations that lacked significant social context, and Telephone Encounters were often brief updates or check-ins that did not provide the depth of information necessary for influencing treatment predictions. Interestingly, Finance, which represents the socioeconomic factor that likely influences patients’ decisions in therapy regimen, did not come up as an important regulator in the process. This could indicate that financial concerns are less frequently or explicitly documented in social work notes compared to other SDOH factors, or that the nuances of financial strain are harder to capture and quantify within the context of these notes. Although further human validation by an external clinician found only one mention of a target therapy drug in 50 randomly selected notes, the overall analysis using model interpretability methods was successful. Our results revealed that SDOH factors, which are not commonly considered influencers in the prescription of financially burdensome oncology medications, do indeed play a role.

## Discussion

This study demonstrated that clinical social work documentation, which focuses on social determinants of health rather than treatment plans, can be predictive of whether targeted therapies are administered to patients with breast cancer and highlights a potential SDOH-dependent disparity in therapy administration. Additionally, we developed a hierarchical modeling technique to incorporate the large volume of note data within any given chart, which often exceeds the processing capacity of the state-of-the-art NLP models. This technique can leverage multiple notes for prediction without adding a significant amount of computation burden. Finally, we performed a feature importance analysis by ablation of SDOH-related keywords to better understand which topics within social work notes have the greatest contribution to model performance.

We found that pretraining a language model on similar data sources is important for better prediction performance in specialized domains, particularly from small datasets that are common in clinical studies. Among all the transformer-based models we explored (Table 1), Gatortron-OG achieved the best prediction performance on our task. Moreover, with our hierarchical BERT model, we showed that integrating multiple notes, and consequently more information about a patient, improves model performance. It is generally accepted that including more comprehensive patient information, either from clinical notes written at different times during a health encounter or for a different purpose, will lead to better performance for prediction tasks. Although alternate methods that allow longer input text exist, such as the Longformer technique,^41^ these approaches usually require retraining a large language model, which can be time-consuming and computationally expensive. In addition, multiple instance learning methods could also potentially serve as more efficient methodologies for integrating multiple notes that normally exceed the size of a regular BERT model.^42^ However, the use of longer contexts may not always be beneficial or needed, especially if a task can be successfully solved with local, short contexts.

In our feature importance analysis, we found that financial factors are not the sole SDOH factors influencing therapy regimen decisions, as initially hypothesized. Our feature importance analysis revealed other significant factors, including “risk of death” and “abuse history,” led to decreases in model performance when removed from social work note text. Additional SDOH topics such as Family and Living Condition could also have an obvious impact on model decision-making. For instance, Family-related notes often included information about the level of support from family members, conflicts within the family, or the presence of caregivers, all of which can significantly influence a patient's treatment adherence and choices. Similarly, notes about Living Conditions, such as descriptions of unstable housing situations, overcrowded living spaces, or frequent relocations, provided crucial context that the model used to predict therapy selections. Notably, simply extracting whether social work topics are present in the given social work notes as input features for classification was insufficient. This demonstrated it is important to consider the context of topic mentions within social work notes (Table S8). This study broadens our understanding of the various factors affecting therapy regimen choices, suggesting that a more comprehensive approach is needed when considering SDOH factors in clinical informatics. Future research should explore additional factors and their potential impact on therapy decisions to ensure a more holistic understanding of patient care.

There are several limitations to this study. While our research showed that social work reports that encompass SDOH information are predictive of the administered breast cancer therapy regimen, integration of structured data and other types of text reports may both highlight other aspects driving the disparity in treatment choice and improve overall predictive performance. While the aim of our paper is to demonstrate the utility of social work notes, comprehensively predicting therapy regimen decisions is complex and beyond the scope of the current paper. In addition, we are limited by the number of breast cancer patient with social work notes, since only a small proportion of patients have social work notes. However, given the focus of our paper, the data selection choice of the study is reasonable. We believe it makes the findings even more intriguing, since the study demonstrates that the administered therapy can be systematically predicted from social work notes even among patient groups who may already have a negative valence SDOH, compared to the entire patient cohort. Another limitation is the difficulty of masking drug information using rule-based systems or keyword searches. While the study demonstrated that similar results could be obtained from notes without drug mentions, simple masking may still cause information leakage. Using a large language model to fully mask drug information before model training could enhance the robustness of our approach. In addition, our study included social work notes from any time point without filtering for their timing relative to therapy initiation, we acknowledge the potential for indirect influences where underlying conditions might bias the topics discussed in these notes. In future studies, we will consider implementing temporal controls to more accurately assess the causal relationships between drug prescriptions and social work consultations.

Moreover, systematically extracting and converting all SDOH factors from clinical notes to structured data may create additional opportunities for further analysis. The driving forces behind treatment decisions for patients at other centers may differ, as may the overall distribution of SDOH factors themselves.^43^ Patients may already be preselected in unrecorded ways to receive a social worker consultation. Future work should seek to integrate data across institutions with differing practices to further validate our findings.

## Conclusion

In conclusion, our study demonstrates the potential of utilizing transformer-based deep learning approaches for predicting clinical outcomes using social work reports. Specifically, our findings indicate the presence of notable disparities in treatment regimens, which can be attributed to social determinants of health. By creating a hierarchical model that can incorporate additional notes, we observed an enhancement in overall model performance. Through the use of ablation methods to better understand model interpretability, we highlighted the variety of SDOH factors that can influence therapy regimen selection for patients with breast cancer. Future research should extend this analysis to explore the impact of SDOH on treatment selection at other institutions and for different types of cancer.

## Supplementary Material

ooae073_Supplementary_Data

## Data Availability

The data that support the findings of this study are available from the Information Commons platform at UCSF, but restrictions apply to the availability of these data, which were used under license for the current study, and so are not publicly available. Data are however available from the authors upon reasonable request and with permission of UCSF.
