# Supplementary material for: Revealing the impact of social circumstances on the selection of cancer therapy through natural language processing of social work notes
Source: JAMIA Open. 2024 Oct 11;7(4):ooae073. doi: 10.1093/jamiaopen/ooae073 (PMC11470153; doi:10.1093/jamiaopen/ooae073)
Supplement: ooae073_Supplementary_Data [file ooae073_supplementary_data.docx]

# **Supplementary materials**

***Supplementary figures***


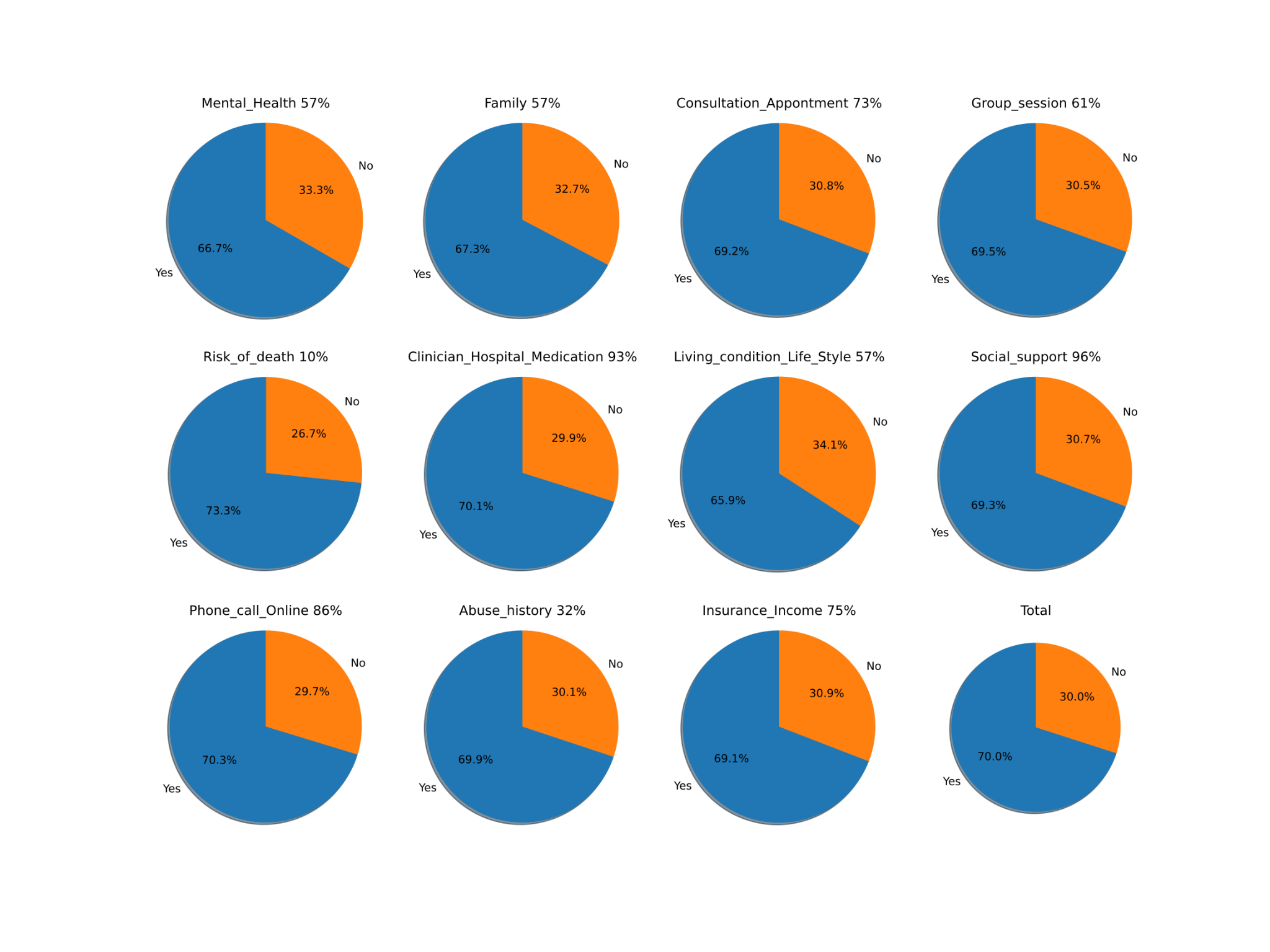


**Supplementary Figure 1.** Pie chart showing the different proportions of patients in the two categories per topic category. The percentage on the right of each topic indicates the frequency of whether words in the topics existed in individual social work notes. Orange: Patients who did not receive any targeted therapy. Blue: Patients who received at least one dose of targeted therapy.


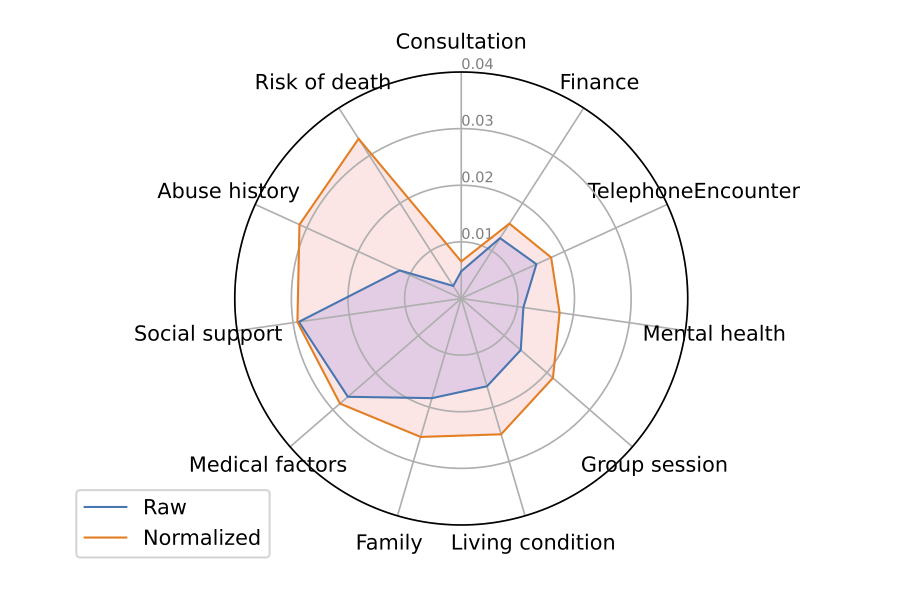


**Supplementary Figure 2**. Feature importance analysis for SDOH factors. The radar chart shows the feature importance of SDOH topics. Feature importance is defined to be the decrease in the F1 score of *Targeted therapy not administered* class across the entire test set overall (Raw: Blue), and across the notes that actually contain these words (Normalized: Orange).


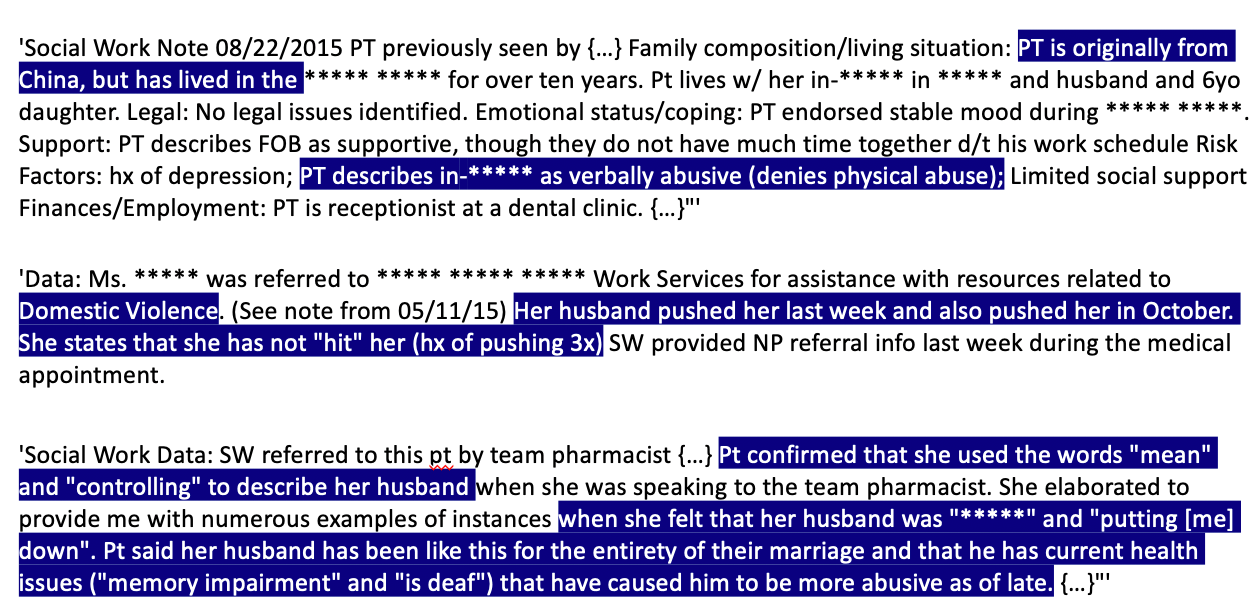


**Supplementary Figure 3**. Example deidentified social work notes contain abusive history information.

***Supplementary algorithm***

Pseudo codes for training BERT-MS model

# *Define the class:*

Class BERT_classification

    Function initialize()

        # Initialize BERT model as the encoder layer

        self.encoder = initialize_BERT_encoder()

        # Initialize fully connected layer for classification

        self.fc_layer = initialize_fully_connected_layer()

    Function forward(x)

        # Get feature embeddings from the encoder

        feature_embedding = self.encoder(x)

        # Get predictions from the fully connected layer

        prediction = self.fc_layer(feature_embedding)

        return prediction

    Function train(data)

Class BERT_MS_classification

    Function __init__(self, BERT_encoder, n)

        # Initialize BERT model as the encoder layer with frozen weights

        self.encoder = BERT_encoder

        freeze_weights(self.encoder)

        # Initialize fully connected layer for classification with n as input

        self.fc_layer = initialize_fully_connected_layer(n)

    Function forward(self, x)

        # Initialize an empty list for concatenated feature embeddings

        concatenated_feature_embeddings = []

        # Iterate over the input list [x1, x2, ..., xn]

        for xi in x:

            # Get feature embeddings from the encoder

            feature_embedding = self.encoder(xi)

            # Append the feature embedding to the concatenated_feature_embeddings list

            concatenated_feature_embeddings.append(feature_embedding)

        # Use the MLP to get the prediction based on concatenated_feature_embeddings

        prediction = self.fc_layer(concatenated_feature_embeddings)

        return prediction

    Function train(data)

*# Training pipeline*

Function train_BERT_MS_n(data, n)

    # Step 1: Fine-tune BERT classification model on individual notes

    # Step 1.1: Create independent instances for training

    instances = create_independent_instances(data)

    # Step 1.2: Initialize BERT model and add a fully connected layer for classification

    Single_step_model = BERT_classification()

    Single_step_model.initialize()

    Single_step_model.train(instances)

    # Step 2: Initialize BERT-MS-classification model

    Multi_step_model = BERT-MS-classification()

    Multi_step_model.initialize(Single_step_model.encoder, n)

    Multi_step_model.train(instances)

# Train various BERT-MS-n models

BERT_MS_3_model = train_BERT_MS_n(data, 3)

BERT_MS_5_model = train_BERT_MS_n(data, 5)

BERT_MS_8_model = train_BERT_MS_n(data, 8)

BERT_MS_10_model = train_BERT_MS_n(data, 10)

***Supplementary tables***

S. Table 1: Demographic characteristics for breast cancer patients in our cohort.

|  | Targeted therapy not administered (TT-No, N=597) | Targeted therapy administered  (TT-Yes, N=1899) | Overall  (N=2496) |
| --- | --- | --- | --- |
| **Sex** |  |  |  |
| Female | 572 (95.8%) | 1871 (98.5%) | 2443 (97.9%) |
| Male | 25 (4.2%) | 28 (1.5%) | 53 (2.1%) |
| **Ethnicity** |  |  |  |
| Hispanic/Latino | 72 (12.1%) | 180 (9.5%) | 252 (10.1%) |
| Not Hispanic or Latino | 497 (83.2%) | 1646 (86.7%) | 2143 (85.9%) |
| Other | 28 (4.6%) | 73 (2.0%) | 101 (2.2%) |
| **Race** |  |  |  |
| Asian | 110 (18.4%) | 341 (18.0%) | 451 (18.1%) |
| Black/African | 33 (5.5%) | 129 (6.8%) | 162 (6.5%) |
| White or Caucasian | 355 (59.5%) | 1136 (59.8%) | 1491 (59.7%) |
| Other | 99 (17.6%) | 283 (15.4%) | 382(15.7%) |

**S. Table 2**: Summary characteristics of social factors (smoking and marital status) for breast cancer patients extracted from structured data

|  | Targeted therapy not administered (TT-No , N=597) | Targeted therapy administered  (TT-Yes, N=1899) | Overall  (N=2496) |
| --- | --- | --- | --- |

| **Smoking status** |  |  |  |
| --- | --- | --- | --- |
| Current Every day Smoker | 11 (1.8%) | 39 (2.1%) | 50 (2.0%) |
| Current Some Day Smoker | 6 (1.0%) | 21 (1.1%) | 27 (1.1%) |
| Former Smoker | 177 (29.6%) | 569 (30.0%) | 746 (29.9%) |
| Never Assessed | 2 (0.3%) | 9 (0.5%) | 11 (0.4%) |
| Never Smoker | 395 (66.2%) | 1232 (64.9%) | 1627 (65.2%) |
| Passive Smoke Exposure - Never Smoker | 5 (0.8%) | 17 (0.9%) | 22 (0.9%) |
| Smoker, Current Status Unknown | 1 (0.2%) | 1 (0.1%) | 2 (0.1%) |
| *Unknown | 0 (0%) | 6 (0.3%) | 6 (0.2%) |
| Light Tobacco Smoker | 0 (0%) | 3 (0.2%) | 3 (0.1%) |
| Unknown If Ever Smoked | 0 (0%) | 2 (0.1%) | 2 (0.1%) |
| **Marital status** |  |  |  |
| *Unspecified | 1 (0.2%) | 0 (0%) | 1 (0.0%) |
| Divorced | 65 (10.9%) | 212 (11.2%) | 277 (11.1%) |
| Legally Separated | 4 (0.7%) | 15 (0.8%) | 19 (0.8%) |
| Married | 286 (47.9%) | 909 (47.9%) | 1195 (47.9%) |
| Registered Domestic Partner | 7 (1.2%) | 9 (0.5%) | 16 (0.6%) |
| Significant Other | 14 (2.3%) | 37 (1.9%) | 51 (2.0%) |
| Single | 164 (27.5%) | 480 (25.3%) | 644 (25.8%) |
| Unknown/Declined | 18 (3.0%) | 43 (2.3%) | 61 (2.4%) |
| Widowed | 38 (6.4%) | 193 (10.2%) | 231 (9.3%) |
| RDP-Widow | 0 (0%) | 1 (0.1%) | 1 (0.0%) |

**S. Table 3.** Model performances of common machine learning classifiers using SDOH related structured tabular data on targeted therapy administration.

|  | **AUC** | **MACRO F1** | **MACRO**  **PRECISION** | **MACRO**  **RECALL** |
| --- | --- | --- | --- | --- |
| KNeighborsClassifier | 0.497 | 0.491 | 0.496 | 0.497 |
| SVM Classifier | 0.500 | 0.434 | 0.383 | 0.500 |
| RandomForestClassifier | 0.519 | 0.483 | 0.592 | 0.517 |
| GradientBoostingClassifier | 0.509 | 0.458 | 0.635 | 0.509 |

**S. Table 4.** The properties of notes for breast cancer patient’s cohort. (Measure the tokes length and compare with 512 tokens, 512 tokens might correspond to about 2500 characters)

| Percentage of notes longer than >2000 characters | Percentage of notes longer than >2500 characters | Percentage of notes longer  than >300 words | Percentage of notes longer  than >400 words |
| --- | --- | --- | --- |
| 0.8% | 0.0% | 23.1% | 2.4% |

| **S. Table 5:** The words in the *Keywords* column are the | | |  |
| --- | --- | --- | --- |
| representative words used to define the topics | | . |  |
| Topics | Keywords | | |
| Family | family, parent, father, mother, child, children, sister, parents, relatives, clan, childhood, friends | | |
| Consultation/Appointment | appointment, consultation, consult, questionnaire, question, advice, biographical, wikipedia, relevant, questions, know, documentation | | |
| Group session | group, intervention, session, interpers, community, class, organization, together, part, organization | | |
| Risk of death | suicide, suicidal, risk, crisis, homicide, murder, commit, bombing, murdered, murders, bomber, killing, convicted, victims | | |
| Medical factors | patient, medication, hospital, medical, clinic, clinician, treatment, therapy, surgery, symptoms, patients, drugs, diagnosis, treatments, prescribed | | |
| Living condition/Lifestyle | shelter, housing, house, living, sleep, bedtime, building, buildings, urban, employment, suburban, campus, acres | | |
| Social support | social, service, support, referral, recommendation, recommend, worker, resource, supports, provide, supporting, supported, allow, providing, assistance, benefit, help | | |
| TelephoneEcounter/Online communication | telehealth, phone, call, video, telephone, mobile, wireless, gsm, cellular, dial, email, calling, networks, calls, messages, telephones, internet | | |
| Abuse history | abuse, history, addiction, alcohol, drugs, allegations, victim, violence, sexual, rape, dependence | | |
| Insurance/Income | insurance, income, coverage, financial, contracts, banking, finance, liability, private, pay | | |

S. Table 6 Model performance of different classifiers. External big language model Gatortron achieved the state-of-the-art performance, demonstrating the reliability of our discovery.

| **Model** | **AUC** | **MACRO F1** | **MACRO**  **PRECISION** | **MACRO**  **RECALL** |
| --- | --- | --- | --- | --- |
| UCSF BERT | 0.675 | 0.599 | 0.604 | 0.596 |
| Gatortron-OG | **0.721** | **0.616** | **0.624** | **0.611** |
| ClinicalBERT | 0.627 | 0.578 | 0.584 | 0.576 |
| SciBERT | 0.616 | 0.532 | 0.606 | 0.533 |
| BioLM | 0.671 | 0.583 | 0.615 | 0.580 |
| Biomed-RoBERTa | 0.667 | 0.584 | 0.592 | 0.581 |
| Dummy (Prior) | 0.500 | 0.412 | 0.350 | 0.491 |
| Dummy (stratified) | 0.504 | 0.525 | 0.529 | 0.603 |
| Dummy (Uniform) | 0.500 | 0.509 | 0.522 | 0.602 |

**S. Table 7. The removal of notes drug mentioning in the prediction pipeline.**

| **Model** | **AUC** | **MACRO F1** | **MACRO**  **PRECISION** | **MACRO**  **RECALL** |
| --- | --- | --- | --- | --- |
| UCSF BERT (with Drug info masked) | 0.675 | 0.599 | 0.604 | 0.596 |
| UCSF BERT excluding notes mentioning Drug | 0.696 | 0.585 | 0.622 | 0.562 |

**S. Table 8. Model performance of leveraging SDOH topics appearance on regimen prediction, without semantic meanings.**

|  | **F1** | **Precision** | **Recall** | **Accuracy** |
| --- | --- | --- | --- | --- |
| **SVM** | 0.408 | 0.345 | 0.500 | 0.690 |
| **Logistic Regression** | 0.408 | 0.345 | 0.500 | 0.690 |
| **Random Forest** | 0.513 | 0.516 | 0.514 | 0.603 |
| **Multilayer perceptron** | 0.456 | 0.598 | 0.51 | 0.690 |
| **Naive Bayes** | 0.401 | 0.406 | 0.399 | 0.466 |

**S. Table 9** Demographic characteristics for all breast cancer patients

|  | Overall  (N=30631) |  |
| --- | --- | --- |
| **Sex** |  |  |
| Female | 30026 (98.0%) |  |
| Male | 589 (1.9%) |  |
| **Ethnicity** |  |  |
| Hispanic/Latino | 1913 (6.2%) |  |
| Not Hispanic or Latino | 23407 (76.4%) |  |
| Other | 5311 (17.4%) |  |
| **Race** |  |  |
| Asian | 2809 (9.2%) |  |
| Black/African | 1411 (4.6%) |  |
| White or Caucasian | 18744 (61.2%) |  |
| Other | 7667 (25.0%) |  |

**S. Table 10**: Summary characteristics of social factors (smoking and marital status) for all breast cancer patients

|  | **Overall**  **(N=30631)** |
| --- | --- |
| **Smokingstatus** |  |
| *Not Applicable | 1 (0.0%) |
| *Unknown | 10416 (34.0%) |
| *Unspecified | 1425 (4.7%) |
| Current Every Day Smoker | 135 (0.4%) |
| Current Some Day Smoker | 43 (0.1%) |
| Every Day | 210 (0.7%) |
| Former | 3336 (10.9%) |
| Former Smoker | 1724 (5.6%) |
| Heavy Smoker | 5 (0.0%) |
| Heavy Tobacco Smoker | 1 (0.0%) |
| Light Smoker | 24 (0.1%) |
| Light Tobacco Smoker | 5 (0.0%) |
| Never | 8053 (26.3%) |
| Never Assessed | 925 (3.0%) |
| Never Smoker | 3533 (11.5%) |
| Passive Smoke Exposure - Never Smoker | 129 (0.4%) |
| Smoker, Current Status Unknown | 36 (0.1%) |
| Some Days | 78 (0.3%) |
| Unknown | 70 (0.2%) |
| Unknown If Ever Smoked | 38 (0.1%) |
| **Maritalstatus** |  |
|  | 3 (0.0%) |
| *Not Applicable | 1 (0.0%) |
| *Unknown | 1 (0.0%) |
| *Unspecified | 35 (0.1%) |
| Divorced | 2506 (8.2%) |
| Legally Separated | 197 (0.6%) |
| Married | 16229 (53.0%) |
| RDP-Dissolved | 3 (0.0%) |
| RDP-LG SEP | 1 (0.0%) |
| RDP-Widowed | 38 (0.1%) |
| Registered Domestic Partner | 74 (0.2%) |
| Significant Other | 225 (0.7%) |
| Single | 6284 (20.5%) |
| Unknown/Declined | 2069 (6.8%) |
| Widowed | 2965 (9.7%) |
|  |  |

**S Table 12. The drug names that we masked.**

carboplatin

intravenous

riTUXimab

carfilzomib

CARBOplatin

trastuzumab-anns

etoposide

brigatinib

hydroxyurea

cisplatin

ruxolitinib

inotuzumab

ibrutinib

irinotecan

ciloleucel

anastrozole

copanlisib

dabrafenib

mechlorethamine

venetoclax

acalabrutinib

procarbazine

larotrectinib

triptorelin

flutamide

mitotane

melphalan

methoxsalen

midostaurin

inFLIXimab

abiraterone

avelumab

cytarabine

vinCRIStine

lapatinib

ifosfamide

fluorouraciL

erlotinib

DAUNOrubicin

DOCEtaxeL

ramucirumab

vinORELBine

thioguanine

alpelisib

decitabine

IDArubicin

encorafenib

capecitabine

trastuzumab

goserelin

pemetrexed

ivosidenib

cladribine

metHOTREXate

SORAfenib

interferon

alemtuzumab

daratumumab

elotuzumab

tamoxifen

mitoMYcin

certolizumab

intrathecal

vemurafenib

bicalutamide

megestroL

axitinib

gemtuzumab

durvalumab

enasidenib

ipilimumab

cabazitaxeL

emtansine

olaparib

PACLitaxeL

temozolomide

hyaluronid

bevacizumab

doxorubicin

bevacizumab

lenvatinib

PEMEtrexed

DOXOrubicin

cabozantinib

osimertinib

mercaptopurine

cetuximab

etanercept

bleomycin

riTUXimab

hyaluronidase

intravesical

deruxtecan

epiRUBicin

siltuximab

epirubicin

everolimus

neratinib

trastuzumab

bevacizumab

enzalutamide

vedotin

gilteritinib

eribulin

panitumumab

fludarabine

vandetanib

topotecan

megestrol

exemestane

niraparib

adalimumab

trifluridine

tipiracil

regorafenib

PAZOPanib

fulvestrant

ceritinib

lenalidomide

afatinib

CISplatin

pomalidomide

laherparepvec

tucatinib

mebutate

sorafenib

clofarabine

thiotepa

bortezomib

ixazomib

fluorouracil

dasatinib

trastuzumab

talimogene

nilotinib

obinutuzumab

cyclophosphamide

sipuleucel-T

panobinostat

carmustine

eriBULin

toremifene

trioxide

govitecan-hziy

diclofenac

dacarbazine

leuprolide

binimetinib

betadex

methotrexate

letrozole

chlorambuciL

rucaparib

pazopanib

atezolizumab

paclitaxel

crizotinib

PACLitaxel-protein

aminolevulinic

pembrolizumab

infliximab

ribociclib

hyaluronidase

SUNItinib

abemaciclib

trametinib

sunitinib

ixabepilone

lorlatinib

sacituzumab

vorinostat

rituximab

paclitaxel

PONATinib

DACTINomycin

azaCITIDine

tretinoin

bexarotene

darolutamide

sulfobutyl

liposomaL

trastuzumab

olaratumab

degarelix

brentuximab

hyaluronidase

entrectinib

palbociclib

trifluridine

pegaspargase

vinorelbine

subcutaneous

alectinib

blinatumomab

oxaliplatin

bendamustine

lomustine

pertuzumab

gefitinib

imatinib

cemiplimab

trabectedin

ozogamicin

talazoparib

axicabtagene

temsirolimus

gemcitabine

golimumab

bosutinib

docetaxel

aldesleukin

nivolumab

cobimetinib

vinBLAStine
